# Effect of Hospital Characteristics on the Quality of Laparoscopic Gastrectomy in Japan

**DOI:** 10.4021/gr2010.04.189w

**Published:** 2010-03-20

**Authors:** Kazuaki Kuwabara, Shinya Matsuda, Kiyohide Fushimi, Koichi B Ishikawa, Hiromasa Horiguchi, Kenji Fujimori

**Affiliations:** a Kyushu University Graduate School of Medical Sciences, Department of Health Care Administration and Management, 3-1-1 Maidashi, Higashi-ku, Fukuoka, Japan 812-8582; b University of Occupational and Environmental Health, 1-1 Iseigaoka Yahatanishi-ku Kitakyushu, Fukuoka 807-8555, Japan; c Tokyo Medical and Dental University, 1-5-45 Yushima Bunkyo-ku, Tokyo 113-8510, Japan; d National Cancer Center, 5-1-1 Tsukiji Chuo-ku, Tokyo 104-0045, Japan; e University of Tokyo Graduate School of Medicine, 7-3-1 Hongo Bunkyo-ku, Tokyo 113-0033, Japan; f Hokkaido University, 5 Nishi 14 Kita Kita-ku, Sapporo, Hokkaido 060-8648, Japan

**Keywords:** Hospital volume, Laparoscopic gastrectomy, Length of stay, Operating time, Quality of care

## Abstract

**Background:**

Laparoscopic gastrectomy (LG) is becoming more widely indicated, although its application has not been investigated sufficiently in community-based gastrointestinal research because the small number of gastric cancers in western countries might have limited its use. However, concerns have been raised regarding variations in the quality of care with LG. To contribute to improving the efficient utilization of costly surgical innovations, we determined the impact of hospital characteristics on LG care.

**Methods:**

Among 3,914 LG patients in 58 academic and 200 community hospitals between 2006 and 2008, we examined patient demographics, comorbidity, complications, partial or total gastrectomy, care process, hospital patient volume, hospital ownership and teaching status, and fiscal year. Hospital LG volume was divided into three quintile categories (lower volume, LV; intermediate volume, IV; or higher volume, HV) that consisted of an approximately equal number of patients. Dependent variables were operating time (OT), length of stay (LOS) and total charge (TC). The impact of hospital characteristics on these variables was assessed using multivariate analysis.

**Results:**

Twenty-seven academic hospitals out of 193 LV hospitals treated 271 (21%) LG patients, 20 of 44 IV hospitals treated 596 (47%), and 11 of 21 HV hospitals treated 748 (55%). Although mortality or complications did not vary significantly between LV, IV and HV hospitals, the latter were associated with longer OT or LOS and more TC. More blood transfusions were required in HV hospitals once indicated. Hospital ownership or teaching status did not explain the variation in complications. Teaching and national hospitals consumed more resources, and municipal and private hospitals reduced OT more than national hospitals.

**Conclusions:**

A volume-quality relationship was recognized. As intraoperative transfusion prolongs OT and results in more complications, clinical societies or policy makers should introduce this new technique concurrently with quality improvement initiatives that aim to reduce unnecessary OT at targeted institutions. Hospitals varied in terms of LOS and TC, therefore, policy makers should also monitor resource utilization to enhance the efficiency of LG care under restrictive fiscal policies.

## Introduction

Gradual confirmation of the safety and superiority of laparoscopic gastrectomy (LG) over conventional open gastrectomy (OG) has expanded the indications for LG in patients with advanced-stage gastric cancer, or those admitted to community hospitals [1-3]. As the estimated annual number of LGs in Japan has increased from around 3,600 in 2006 to 9,600 in 2007, these cases have provided cumulative clinical and claims data, which have enabled quantitative analysis of the quality of LG, and identification of hospital characteristics associated with quality of LG care, through an administrative database [4, 5].

Several studies have evaluated factors that affect the utilization of technical innovations, including laparoscopic surgery or diagnostic imaging [6-11]. Hospital factors such as volume, geographic region, ownership, teaching status, or socioeconomic status have been observed to explain variations in the quality of surgical innovation. Population studies using the administrative database have contributed toward those observations.

However, application of LG has not been investigated sufficiently in community-based studies because the smaller number of gastric cancers in western countries might have limited identification of variation in LG use [7]. As LG use has increased in Japan, community-based studies of variation in quality of care should be carried out by focusing on hospital characteristics. The findings derived from such studies could contribute to improving the utilization of costly surgical innovations such as LG, and policy-makers could play a definitive role in quality improvement initiatives through implementation of restrictive fiscal policies.

In this study, we determined the impact of hospital characteristics on complications, resource use and care of LG patients in Japan.

## Materials and Methods

We used a Japanese administrative database that was incorporated into the Ministry of Health, Labor and Welfare (MHLW) and our own research project that was concerned with the development of a Japanese case-mix classification system. Up to 82 academic and 1,346 community hospitals were enrolled in this project in 2008. Anonymous health insurance claims data with detailed clinical information have been collected annually for a study period of 6 months from July 1, 2006 for this database, and were provided to our research team. This database contained details of the date and quantity of care provided during hospitalization, therefore, it was used to assess hospital performance and payments by MHLW. Among a total of 8,010,361 patients from 1,006 hospitals that voluntarily participated in our research project, we analyzed gastrectomy patients treated consecutively in 258 hospitals from fiscal year (FY) 2006 to 2008. Our project was approved by the ethical committee of the University of Occupational and Environmental Health, Fukuoka, Japan.

### Variable definitions

Study variables included age, sex, discharge outcome, pathology of gastric disease (benign or malignant), comorbidity, physical status classification by the American Society of Anesthesiologists (ASA), gastrectomy type (partial or total gastrectomy), procedure-related complications, chemotherapy, care process (fasting day, day of postoperative epidural anesthesia, blood transfusion, use or number of circular or linear stapling devices fired), hospital LG volume, hospital ownership and teaching status, and FY. We also calculated length of stay (LOS), total charges (TCs; US$1= ¥100) and operating time (OT). TCs included fees for physician consultation and administration, and costs of instruments, laboratory tests and imaging. TC, which is billed during admission, is considered to be a good estimate of healthcare costs [12]. OT included the induction of general anesthesia and preparation for video-monitoring and surgical procedures, in addition to skin to skin time.

Patients were stratified into two age groups: < 65 years, and ≥ 65 years. Diagnosis was coded by the International Classification of Disease 10th version (ICD code). Malignant diseases were referred to as C16 and C170, and benign diseases as D131-2, D371, K25-6, and K31. The database recorded a maximum of either four comorbidities or four complications per patient.

To assess the severity of preexisting comorbid conditions, we used the Charlson comorbidity index (CCI) [13]. Patients were divided into four groups: CCI = 0, 1, 2 or ≥ 3. ASA score was summarized into three groups: 1, 2 or ≥ 3. Procedure-related complications included wound complications, hematoma or others (T81 - T87), bowel obstruction (K565-7 and K913), and acute pancreatitis (K85) [14]. Use of chemotherapy or preoperative blood transfusion was a proxy for disease severity, advanced gastric cancer or preexisting anemia. Gastrectomy type was recorded by the Japanese original claim code for which gastrectomy was categorized as partial or total or as OG or LG.

Hospital LG volume was divided into three quintile categories (lower volume, LV; intermediate volume, IV; or higher volume, HV) that consisted of an approximately equal number of patients. Hospital ownership was classified into four groups (national, municipal, non-profit private, and private for profit). Non-profit private hospitals included Red Cross Hospitals and charity hospitals. The remaining private hospitals were designated as private for profit (Japanese legislation prohibits them to have stock holders). Teaching hospitals were responsible for the clinical research as well as education of medical students and postgraduate trainees.

### Statistical analysis

Frequencies and proportions for categorical data were compared by Fisher’s exact test. Continuous variables were compared between LV, IV and HV hospitals using analysis of variance. Logistic regression was used to evaluate the association of hospital characteristics and volume with complications. Multiple linear regression analysis was employed to assess the effect of hospital variables on OT, LOS and TC. Statistical analysis was performed using SPSS version 16.0, with a level of significance set at p < 0.05.

## Results

The 58 academic hospitals (27 LV, 20 IV and 11 HV) and 200 community hospitals (166 LV, 24 IV and 10 HV) included a total of 3,914 LG patients. LV hospitals performed a mean 4.5 LG procedures annually (range 0.7 - 12.7); IV hospitals performed a mean 19.1 (13.3 - 25.3); and HV hospitals performed a mean 43.2 (26 - 106.7). As for ownership, 40 national hospitals (22 LV, 11 IV and 7 HV) provided care for a mean 14.5 LG patients annually (range 0.7 - 52.0); 52 municipal hospitals (41 LV, 7 IV and 4 HV) cared for 8.6 (0.7 - 43.3); 79 non-profit private hospitals (67 LV, 9 IV and 3 HV) cared for 7.7 (0.7 - 48.7); and 87 private for profit hospitals (63 LV, 17 IV and 7 HV) cared for 11.1 (0.7 - 106.7) patients.

There was no significant difference in age, sex, mortality and complications between hospital volume categories. Patients with less comorbidity or more malignant disease, and more indications for total gastrectomy were observed in HV hospitals. Private hospitals used LG more than national and municipal hospitals. As FY progressed, more LG was indicated in every volume category (Table 1).

**Table 1:** Patient or hospital characteristics, care process and outcomes by hospital volume category (n, %).

		LVH	IVH	HVH	P
Overall		1291	1261	1362	
Age (y)	Mean, [SD]	64.2 [11.7]	65.0 [11.5]	64.9 [11.5]	<0.148†
	65≤	658 (51.0)	700 (55.5)	740 (54.3)	0.057
Gender					
	Male	804 (62.3)	813 (64.5)	903 (66.3)	0.096
Outcome					
	Mortality	5 (0.4)	3 (0.2)	5 (0.4)	0.776
CCI					
	1	238 (18.4)	244 (19.3)	177 (13.0)	<0.001
	2	83 (6.4)	99 (7.9)	90 (6.6)	
	3≤	39 (3.0)	55 (4.4)	35 (2.6)	
Principal diagnosis					
	Malignancy	1111 (86.1)	1152 (91.4)	1286 (94.4)	<0.001
ASA					
	1	1165 (90.2)	1191 (94.4)	1253 (92.0)	0.001
	2	114 (8.8)	68 (5.4)	103 (7.6)	
	3≤	12 (0.9)	2 (0.2)	6 (0.4)	
Gastrectomy					
	Partial	1206 (93.4)	1134 (89.9)	1158 (85.0)	<0.001
	Total	85 (6.6)	127 (10.1)	204 (15.0)	
					
Complication	136 (10.5)	151 (12.0)	138 (10.1)	0.286	
Ownership					
	National	212 (16.4)	311 (24.7)	349 (25.6)	<0.001
	Municipal	253 (19.6)	201 (15.9)	219 (16.1)	
	Non profit private	487 (37.7)	225 (17.8)	202 (14.8)	
	Private for profit	339 (26.3)	524 (41.6)	592 (43.5)	
Teaching status					
	Community	1020 (79.0)	665 (52.7)	614 (45.1)	<0.001
	Teaching	271 (2.01)	596 (47.3)	748 (54.9)	
FY					
	2006	323 (25.0)	379 (30.1)	360 (26.4)	0.002
	2007	410 (31.8)	429 (34.0)	467 (34.3)	
	2008	558 (43.2)	453 (35.9)	535 (39.3)	

† ; Compared using analysis of variance. Other comparisons made using Fisher's exact test.

LVH; low volume hospital. IVH; intermediate volume hospital. HVH; high volume hospital.

ASA; American Society Anesthesiologist.

The average number of operative/postoperative or overall blood transfusions was greater in HV hospitals, although the proportion of transfusions did not differ significantly among the hospital volume categories. The number of patients undergoing chemotherapy and postoperative pain control was lower in HV hospitals, whereas the proportion of linear stapling devices fired was higher. Postoperative fasting days and OT were shorter in HV hospitals. LOS and TC were significantly greater in LV hospitals, except for TC during hospitalization (p = 0.334) (Table 2).

**Table 2:** Care procedures and resource use by hospital volume category.

	LVH	IVH	HVH	P
Care process				
Chemotherapy	29 (2.2)	14 (1.1)	12 (0.9)	0.006*
Blood transfusion				
preoperative, n (%)	18 (1.4)	10 (0.8)	19 (1.4)	0.271
preoperative ml, mean [SD]	1611.1 [1365.9]	1520.0 [1174.5]	3010.5 [5019.7]	0.336
operative or postoperative, n (%)	51 (4.0)	40 (3.2)	41 (3.0)	0.363
operative or postoperative ml, mean [SD]	1431.4 [1005.1]	1130.0 [874.2]	2351.2 [3525]	0.027
overall n (%)	62 (4.8)	45 (3.6)	57 (4.2)	0.298
overall ml, mean [SD]	1645.2 [1298.9]	1342.2 [1142.3]	2694.7 [4105.4]	0.022
Postoperative fasting period				
days [SD]	5.1 [3.9]	4.7 [3.0]	4.4 [3.3]	<0.001
Use of pain control				
n (%)	1024 (79.3)	1015 (80.5)	1017 (74.7)	0.001
day [SD]	3.0 [1.9]	2.7 [1.3]	3.0 [3.1]	<0.001
Use of circular stapling devices n (%)	601 (46.6)	591 (46.9)	660 (48.5)	0.572
Number of circular stapling devices fired	1.1 [0.2]	1.1 [0.2]	1.1 [0.2]	0.829
Use of linear stapling devices n (%)	1057 (81.9)	1042 (82.6)	1196 (87.8)	<0.001
Number of linear stapling devices fired	2.5 [1.1]	2.5 [0.9]	2.5 [1.0]	0.986
Resource use				
OT, min [SD]	338.3 [113.1]	361.3 [108.1]	337.3 [97.4]	<0.001
Postoperative LOS, days [SD]	18.4 [11.9]	16.6 [10.9]	15.9 [10.2]	<0.001
LOS, days [SD]	23.3 [15.2]	21.3 [13.0]	20.1 [11.7]	<0.001
Postoperative TC, $ [SD]	4558.5 [4389.5]	4087 [3615]	4204.9 [4843.8]	0.016
TC, $ [SD]	14464.2 [6184.1]	14211 [5026.2]	14527.4 [5934.3]	0.334

SD; standard deviation. *;compared using Fisher's exact test. Other comparisons made using analysis of variance.

Longer OT and lower hospital volume were found to be significant predictors of more complications, for which hospital ownership and teaching status were not significant determinants. FY 2008 was associated significantly with more complications (Table 3).

**Table 3:** Factors associated with complication.

	Complication	
	Odds ratio	[ 95% CI ]
Age (y)		
<65	1.000	
65≤	1.322	[1.072 - 1.632]
Sex		
Female	1.000	
Male	1.052	[0.845 - 1.311]
CCI		
absent	1.000	
1	1.654	[1.287 - 2.127]
2	1.304	[0.891 - 1.907]
3≤	2.055	[1.292 - 3.268]
Principal diagnosis		
Benign	1.000	
Malignancy	0.950	[0.645 - 1.398]
ASA		
1	1.000	
2	1.244	[0.855 - 1.810]
3≤	1.214	[0.345 - 4.275]
Chemothrapy		
absent	1.000	
present	0.800	[0.315 - 2.034]
Preoperative blood transfusion		
absent	1.000	
present	0.792	[0.304 - 2.065]
Gatsrectomy		
Partial gastrectomy	1.000	
Total gastrectomy	1.188	[0.862 - 1.638]
OT		
one more minute	1.002	[1.001 - 1.003]
Postoperative pain control		
absent	1.000	
present	0.834	[0.654 - 1.062]
Hospital patient volume		
one more case	0.997	[0.994 - 0.999]
Ownership		
National	1.000	
Municipal	1.059	[0.709 - 1.584]
Non profit private	0.963	[0.645 - 1.438]
Private for profit	1.264	[0.945 - 1.690]
Teaching status		
Community	1.000	
Teaching	1.250	[0.933 - 1.674]
FY		
2006	1.000	
2007	1.003	[0.761 - 1.322]
2008	1.304	[1.009 - 1.684]
HL goodness of model fit.	0.838	

CI; Confidence interval. ***; not included in the model.

ASA; American Society Anesthesiologist. HL; Hosmer Lemeshow.

Lower patient volume and national hospitals were associated significantly with longer OT. Higher patient volume, municipal or private for profit hospitals were significant determinants of shorter LOS and lower TCs, whereas academic hospitals had longer LOS and higher TCs. FY was not a determinant of variation in OT and TC (Table 4).

**Table 4:** Factors associated with operating time (OT), length of stay (LOS) and total charge (TC).

	OT (min)			LOS			TC		
	Estimation	95% CI	P	Estimation	95% CI	P	Estimation	95% CI	P
Intercept	234.3	8.0	<0.001	18.1	1.0	<0.001	9724	406	<0.001
Age	-4.9	3.1	0.117	2.3	0.4	<0.001	828	157	<0.001
Male	25.1	3.2	<0.001	1.1	0.4	0.006	805	163	<0.001
CCI (for zero)									
1	8.3	4.2	0.048	2.8	0.5	<0.001	715	212	0.001
2	18.5	6.1	0.003	2.6	0.8	0.001	982	311	0.002
3 ≤	24.2	8.7	0.005	3.3	1.1	0.002	1572	439	<0.001
Principal diagnosis									
Malignancy	116.0	5.4	<0.001	1.8	0.7	0.009	2132	272	<0.001
ASA (for 1)									
2	5.9	6.0	0.326	0.9	0.8	0.244	287	306	0.348
3–5	4.7	21.5	0.826	-4	2.7	0.142	-786	1090	0.471
Chemotherapeutic agent									
administered	12.6	13.0	0.331	14.6	1.6	<0.001	4444	659	<0.001
Preoperative blood transfusion									
Present	-21.4	14.3	0.135	20.6	1.8	<0.001	7490	725	<0.001
Gastrectomy (for partial gastrectomy)									
Total gastrectomy	74.2	5.0	0.000	5.3	0.6	<0.001	4843	254	<0.001
Postoperative pain control									
Present	4.0	3.8	0.283	-0.6	0.5	0.207	311	191	0.103
Complication									
Present	***	7.0	0.6	<0.001	2535	250	<0.001		
Operative/postoperative blood transufusion†									
Present	71.1	11.4	<0.001	16.2	1.1	<0.001	9728	439	<0.001
Hospital patient volume									
one more case	-0.251	0.050	<0.001	-0.034	0.006	<0.001	-6	3	0.015
Ownership (for national)									
Municipal	-31.7	6.0	<0.001	-1.5	0.8	0.050	-726	306	0.018
Non profit private	-21.8	5.9	<0.001	-0.1	0.7	0.938	-104	301	0.729
Private for profit	-17.8	4.5	<0.001	-1.5	0.6	0.007	-157	229	0.494
Teaching status									
Academic	5.8	4.6	0.203	1.8	0.6	0.002	851	232	<0.001
FY (for 2006)									
2007	4.3	3.9	0.274	-0.9	0.5	0.058	111	200	0.579
2008	-1.5	3.8	0.696	-2.6	0.5	<0.001	196	193	0.311

F test for the model. P<0.001. Coefficient of determination; OT 0.207, LOS 0.207, TC 0.297.

† ; operative blood transfusion in operating day. ***; not included in the regression model. ASA; American Society Anesthesiologist.

## Discussion

This study revealed that higher-volume hospitals achieved more efficient LG care, as well as fewer complications. Significant variation in OT and resource use was observed in relation to hospital ownership and teaching status, however, rate of complications was not so affected. FY was another independent predictor of complications.

There have been various kinds of innovations in laparoscopy-assisted surgery; newer techniques like laparoscopic nephrectomy, adrenalectomy or LG in the present study on the one hand, and more established procedures like laparoscopic cholecystectomy on the other hand, which has been employed for more complicated cases as a result of its proven safety record [7, 10, 11, 15]. Once the feasibility, safety and novelty of these procedures had been confirmed, clinical societies attempted to make them widely available by promoting surgical skills or encouraging their application through health policy initiatives.

To the best of our knowledge, previous studies have not been conducted in western countries because of the relative infrequency of gastric cancer compared with colorectal malignancies [7]. Reid-Lombard et al have discussed the volume-quality relationship in gastrectomy and have supported evidence-based hospital transfer, for which high gastrectomy volume, irrespective of LG or OG, was designated as > 9 to > 50 cases per year [7]. This corresponded to LV to HV hospitals in our study. Even though we acknowledge the difference in the definition of hospital categories, volume-quality relationships in terms of complications and resource use should be addressed as a worldwide concern. HV hospitals did demonstrate superiority in terms of complication rates and efficient care of LG patients over LV and IV hospitals. However, teaching hospitals were associated with greater resource use, but not with complications or longer OT. This might have been because teaching hospitals attempted to provide education in the skills needed for LG by offsetting this against the cost of providing efficient LG care [16]. HV hospitals required a greater number of operative/postoperative blood transfusions, although the frequency of transfusion was lower than that in LV or IV hospitals. The need for more blood transfusions might have arisen from intractable bleeding from splenic tears, which resulted in longer operations and a greater frequency of complications, because operative transfusion proxy of bleeding proved to prolong OT (Table 4). This is one reason why quality improvement initiatives or perioperative multidisciplinary care should be progressed. The appropriate application of stapling devices to prevent accidental bleeding, or a curriculum to educate surgeons and their staff teams about more practical handling devices should be considered [17, 18].

When studying the dissemination of technological innovations like magnetic resonance imaging (MRI) and laparoscopic surgery, or variations in their indications, hospital characteristics and socioeconomic status have always been included in the analytical model [6-11]. Among these, hospital or surgeon volume, and hospital ownership and teaching status have been well documented [8, 19]. In our study, private hospitals were not inclined to make the resources available, although they were able to reduce OT and not increase the rate of complications. Hahm et al have reported that the ownership relationship is significant for MRI [8]. They wish to enhance the reputation of LG by establishing its safety and efficacy, and developing more costly infrastructure, including video-monitoring, skilled staff and disposable devices, this is another justification for implementation of an efficient fiscal policy. Municipal hospitals consumed fewer resources in the present study; therefore, accountability for greater resource spending might be requested in private or academic hospitals [7-11].

There are several limitations to the present study. First, the study period was limited to only 6 months, which might diminish the generalization of our results. Our study sample size, which was representative of one third to half of LG procedures performed in Japan, assured the validity of our study, if we include all hospitals that participated in our project. The exhaustive MHLW database, for which the study period is planned to be extended by up to 12 months in 2010, should also overcome this limitation. Second, LOS in Japan remains generally 2 - 3 times longer than that in Western countries [20]. Japanese hospitals generally provide wound management and nursing home services in addition to acute medical care. Accordingly, our results might actually reflect the real costs incurred during the entire care process [21]. Third, some clinical information such as body mass index (BMI), cancer staging, and extent of lymphadenectomy, was not recorded. Organization for Economic Co-operation and Development (OECD) health data for 2002 have demonstrated that mean BMI was ≤ 23.4 in East Asian people [20], which should not have affected the results of the present study. Administration of chemotherapy was defined as a marker of advanced cancer stage. More complications were found in FY 2008, and extended lymphadenectomy, which is often considered to be associated with postoperative complications, including intraoperative bleeding sufficiently severe to require intraoperative/postoperative blood transfusion, might be more undertaken during LG [22]. Cancer registry, including the extent of lymphadenectomy, accompanied by this administrative database could measure the effect of this clinical information on resource use and the care process, and enhance the findings of the volume-quality relationship.

In conclusion, our analysis demonstrated that hospital patient volume was associated with fewer complications, shorter LOS and lower TCs. Teaching hospitals were associated with longer OT and LOS and higher TCs. Private hospitals did not reduce the resource use in spite of the ability to perform shorter operations. The number of transfusions was lower in HV hospitals, but they required more transfusion once indicated. Intraoperative transfusion prolonged OT, which possibly resulted in more complications. Therefore, clinical societies or policy makers should endeavor to introduce this new technique concurrently with quality improvement initiatives aimed at the targeted institutions, to enhance the efficiency of LG care, and encourage the development of surgical skills to prevent unexpected bleeding.
